# High Expression of LTBP2 Contributes to Poor Prognosis in Colorectal Cancer Patients and Correlates with the Mesenchymal Colorectal Cancer Subtype

**DOI:** 10.1155/2019/5231269

**Published:** 2019-03-10

**Authors:** Ying Huang, Guihua Wang, Chunmei Zhao, Rong Geng, Shu Zhang, Wei Wang, Jing Chen, Huimin Liu, Xudong Wang

**Affiliations:** ^1^Department of Laboratory Medicine, Affiliated Hospital of Nantong University, 20 Xisi Road, Nantong, 226001 JS, China; ^2^Department of Cell Biology, School of Basic Medicine, Nanjing Medical University, Nanjing, 211166 JS, China; ^3^Department of Pathology, Affiliated Hospital of Nantong University, 20 Xisi Road, Nantong, 226001 JS, China; ^4^Clinical Biobank, Affiliated Hospital of Nantong University, 20 Xisi Road, Nantong, 226001 JS, China

## Abstract

Colorectal cancer (CRC) is a complex and heterogeneous disease with four consensus molecular subtypes (CMS1-4). LTBP2 is a member of the fibrillin/LTBP super family and plays a critical role in tumorigenesis by activating TGF-*β* in the CMS4 CRC subtype. So far, the expression and prognostic significance of LTBP2 in CRC remains obscure. In this study, we aimed to analyze the mRNA and protein expression levels of LTBP2 in CRC tissues and then estimate their values as a potential prognostic biomarker. We detected the mRNA expression of LTBP2 in 28 cases of fresh CRC tissues and 4 CRC cell lines and the protein expression of LTBP2 in 483 samples of CRC tissues, matched tumor-adjacent tissues, and benign colorectal diseases. LTBP2 protein expression was then correlated to patients' clinical features and overall survival. Both LTBP2 mRNA and protein expression levels in CRC tissues were remarkably superior to those in adjacent normal colorectal tissues (*P* = 0.0071 and *P* < 0.001, respectively), according to TCGA dataset of CRC. High LTBP2 protein expression was correlated with TNM stage (*P* < 0.001), T stage (*P* < 0.001), N stage (*P* < 0.001), and M stage (*P* < 0.001). High LTBP2 protein expression was related to poor overall survival in CRC patients and was an independent prognostic factor for CRC. LTBP2 mRNA expression was especially higher in the CMS4 subtype (*P* < 0.001), which was confirmed in CRC cell lines. Our data suggested that LTBP2 may act as an oncogene in the development of colorectal cancer and have important significance in predicting CRC prognosis. LTBP2 could be a novel biomarker and potential therapeutic target for mesenchymal colorectal cancer and can improve the outcome of high-risk CRC.

## 1. Introduction

Colorectal cancer (CRC) demonstrates extremely heterogeneous and complex gastrointestinal malignancy, with increasing incidence and mortality according to the newest statistical survey [1]. Despite the remarkable advances in the management of CRC in recent years, the 5-year overall survival (OS) rate is still poor [2, 3]. General thinking suggests that genetic alterations, including somatic gene mutation, deletion, or amplification, copy number variation, and epigenetic modifications, facilitate initiation and progression of CRC. Through the integrated analysis of transcriptomic data, CRC was divided into four subtypes named consensus molecular subtypes (CMS), with the following distinguishing molecular features: CMS1 (MSI immune, 14%); CMS2 (canonical, 37%); CMS3 (metabolic, 13%); and CMS4 (mesenchymal, 23%) [4–6]. Of these, CMS4 is closely related to recurrence and metastasis, and the underlying mechanisms include transforming growth factor *β* (TGF-*β*) activation, stromal invasion, and angiogenesis. It is usually at a relatively later tumor stage when it is diagnosed, and it contributes to worse relapse-free and overall survival rate [7]. Thus, CMS4 has been considered as the worst type of CRC outcome. Early diagnosis and subtype identification can significantly increase the OS rate of CRC; thus, it is urgent for us to find a novel and reliable biomarker for CRC which can improve the outcome of patients with CRC.

Latent transforming growth factor *β* binding protein 2 (LTBP2), a secreted extracellular matrix (ECM) protein, is a member of the fibrillin/LTBP super family, which contains LTBPs 1-4 and fibrillins 1, 2, and 3 [8, 9]. These proteins consist mainly of cysteine-rich EGF-like and 8-cysteine (8-Cys) repeats and share a similar overall domain structure. LTBPs 1, 3, and 4 regulate the biological activities of TGF-*β* family growth factors by covalently binding TGF-*β* and directing the growth factor to storage depots within the extracellular matrix, while LTBP2 is hypothesized to indirectly regulate the activation of TGF-*β* by competing with LTBP1 for the same binding site to fibrillin 1 in microfibrils [10, 11]. Accumulating evidence shows that LTBPs play important roles in tumorigenesis, especially LTBP2. A recent report demonstrated that LTBP2 was involved in the signaling pathway of the mesenchymal subtype in colorectal cancer [12]. However, the clinical implication of LTBP2 expression in CRC remains unknown.

In this study, we determined both the mRNA and protein expression levels of LTBP2 in CRC tissues and matched tumor-adjacent tissues by quantitative real-time polymerase chain reaction (qRT-PCR) and tissue microarray immunohistochemistry (TMA-IHC) analyses, respectively. Then, we used TCGA database and CRC cell lines to confirm our results. Finally, we correlated LTBP2 protein expression to patients' clinical characteristics and estimated its potential prognostic significance.

## 2. Materials and Methods

### 2.1. Tissue Samples and Patients' Clinical Information

A total of 511 patients were included in the study. They provided 56 fresh surgical samples, including 28 cancer tissues and 28 matched adjacent tissues, and 483 archived formalin-fixed paraffin-embedded (FFPE) tissue blocks, including 204 cancer tissues, 190 matched normal surgical margins, 23 chronic colitis tissues, 44 low-grade intraepithelial neoplasia (LIN) tissues, and 22 high-grade intraepithelial neoplasia (HIN) tissues. The 56 fresh surgical samples were received from the Affiliated Hospital of Nantong University between 2016 and 2017. The 483 FFPE tissue blocks were obtained between 2004 and 2014 and were used to construct the TMA using the Tissue Microarray System (Quick-Ray, UT06, Unitma Co. Ltd., Korea). All clinical features containing gender, age, tumor location, differentiation grade, TNM stage, local invasion, regional lymph node metastasis, distant metastasis, and preoperative serum carcinoembryonic antigen (CEA) level were obtained from patients' medical records. All of these patients did not receive any therapy, such as radiotherapy, chemotherapy, and immunotherapy, prior to surgery. Patients were followed up for more than 5 years. This study was approved by the Human Research Ethics Committee of the Affiliated Hospital of Nantong University.

### 2.2. Cell Lines and Cell Culture

Human CRC cell lines (DLD1 and HT29 non-CMS4 subtypes and SW620 and Caco2 CMS4 subtypes) and a normal colorectal epithelial cell line (NCM460) were purchased from the Chinese Academy of Sciences (Shanghai, China). HT29, Caco2, DLD1, SW620, and NCM460 were maintained in McCoy's 5A medium (Gibco, Grand Island, NY, USA), RPMI 1640 medium (Corning, VA, USA), and DMEM medium (Corning, VA, USA) supplemented with 10% fetal bovine serum (FBS, Gibco, CA, USA) and 1% Penicillin/Streptomycin (Gibco, BRL), respectively. All of them were cultured in 5% CO_2_ atmosphere at 37°C. All media were changed every 2-3 days until 90% confluent, and cultures were split using 0.25% trypsin (Gibco, Canada).

### 2.3. RNA Extraction and qRT-PCR Analysis

Total RNA was isolated from CRC tissues and cell lines using the TRIzol Reagent (Invitrogen, CA, USA), and the cDNA was generated using SuperScript® III Reverse Transcriptase (Invitrogen, CA, USA). LTBP2 mRNA levels were measured by qRT-PCR. Relative quantification of mRNA was performed using the ΔΔCt method by first normalizing to the housekeeping gene GAPDH mRNA level and then normalizing to the reference sample. The reverse transcription conditions were 60 min at 42°C and 5 min at 72°C, and the conditions were saved at 4°C. For qRT-PCR, the conditions were 5 min at 95°C followed by 40 cycles of 95°C for 15 s, 60°C for 32 s, and 72°C for 30 s. The primers used are as follows: LTBP2 forward primer (5′-TTACAAGCAGAGACTCACT-3′) and LTBP2 reverse primer (5′-ACAACAGAAGAGACCAGAT-3′) and GAPDH forward primer (5′-GGACCAATACGACCAAATCCG-3′) and GAPDH reverse primer (5′-AGCCACATCGCTCAGACAC-3′).

### 2.4. Immunohistochemistry

LTBP2 protein expression in 483 tissue blocks was examined using IHC performed following the standard protocol [13]. After antigen retrieval, LTBP2 was detected by a rabbit polyclonal anti-human LTBP2 antibody (dilution 1 : 800, ab121193, Abcam, USA) and then incubated with horseradish peroxidase-conjugated goat anti-rabbit antibody (Abcam). The color was developed using 3,3′-diaminobenzidine (Dako, Carpinteria, CA, USA), counterstaining with hematoxylin. The LTBP2 protein expression level was quantified by a two-level grading system using an Olympus BX53 microscope (Olympus Co., Tokyo, Japan). LTBP2 staining intensity was scored as follows: 0 (−, no staining), 1 (+, mild staining), 2 (++, medium staining), or 3 (+++, intense staining). The percentage of positively stained cells (0-100%) was multiplied by the intensity score to give the final IHC score, which ranged from a minimum of 0 to a maximum of 300. The X-tile software program (Rimm Lab at Yale University; http://www.tissuearray.org/rimmlab) was used to determine the cutoff value for low/high protein expression of LTBP2.

### 2.5. Statistical Analysis

SPSS version 20.0 software (SPSS Inc., Chicago, IL, USA) was used for data analysis. The Pearson *χ*^2^ test was applied to evaluate the relationships between the protein expression of LTBP2 and the patients' clinical features. Kaplan-Meier survival curves and the log rank test were used for survival analysis and survival curve drawing. Univariate and multivariate Cox regression analyses were applied to assess the potential prognostic value of LTBP2 protein expression in CRC. *P* values less than 0.05 were considered to be statistically significant.

### 2.6. Bioinformatic Analysis of TCGA Database

The preprocessed level 3 RNA-seq data of colorectal cancer patients were collected from The Cancer Genome Atlas (TCGA) database (http://cancergenome.nih.gov).

## 3. Results

### 3.1. LTBP2 mRNA Expression in CRC Tissues

**Figure 1:**
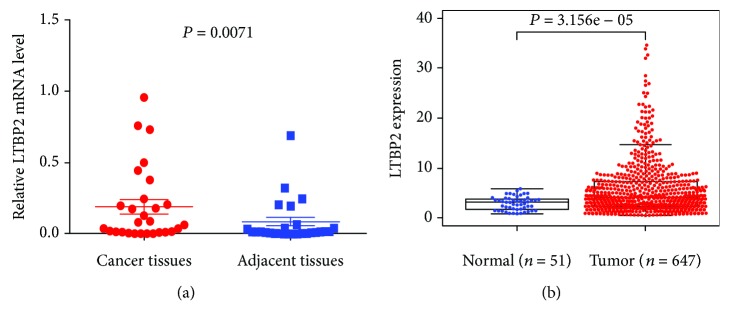
LTBP2 mRNA expression in CRC tissues and cell lines. (a) LTBP2 mRNA expression was significantly higher in CRC tissues than in matched adjacent tissues. LTBP2 mRNA was detected by qRT-PCR, and relative quantification analysis was normalized to GAPDH mRNA (*P* = 0.0071). (b) In TCGA database, LTBP2 mRNA expression was also higher in CRC tissues than in normal colorectal tissues (*P* < 0.0001).

We detected LTBP2 mRNA expression in 28 fresh CRC tissues and 28 matched adjacent tissues by qRT-PCR. LTBP2 mRNA expression was significantly higher in CRC tissues than in matched adjacent tissues (*P* = 0.0071, Figure 1(a)), in accord with TCGA database (*P* < 0.0001, Figure 1(b)).

### 3.2. LTBP2 Protein Expression in CRC Tissues

**Figure 2:**
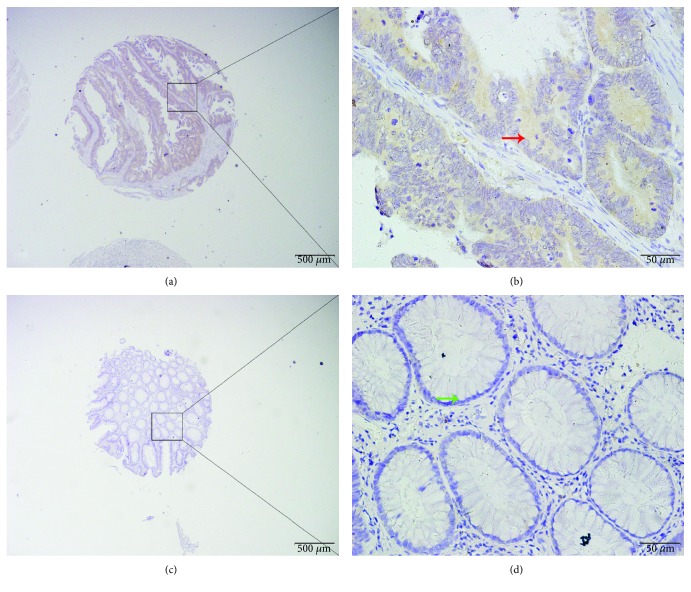
LTBP2 protein expression in CRC tissues. LTBP2 protein was determined by TMA-IHC. (a, b) Colorectal cancer with strong positive LTBP2 protein expression. (c, d) Adjacent normal tissue with negative LTBP2 protein expression. Red arrow represents positive LTBP2 protein expression in CRC tissue, and green arrow represents negative LTBP2 protein expression in adjacent normal tissue. Original magnification is ×40 (bar = 500 *μ*m) in (a) and (c) and ×400 (bar = 50 *μ*m) in (b) and (d).

We determined LTBP2 protein expression in 483 archived tissue blocks including 204 cancer tissues, 190 matched normal surgical margins, 23 chronic colitis tissues, 44 low-grade intraepithelial neoplasia tissues, and 22 high-grade intraepithelial neoplasia tissues. The positive staining of LTBP2 was mainly localized in the cytoplasm of tumor cells (Figure 2). The X-tile software program was used to select the optimal cutoff value (180) for the low/high protein expression of LTBP2, which means that the scores from 0 to 180 and from 181 to 300 were regarded as low and high expressions, respectively. High LTBP2 protein expression was detected in 28.4% of CRC tissues, which was significantly higher compared with the expression detected in 4.3% of chronic colitis tissues, in 6.8% of low-grade intraepithelial neoplasia tissues, in 22.7% of high-grade intraepithelial neoplasia tissues, and in 6.8% of the surgical margin (Pearson *χ*^2^ = 39.896, *P* < 0.001) (Table 1).

### 3.3. Association between LTBP2 Protein Expression and Clinical Features in CRC Patients

The association between LTBP2 protein expression and clinical features in CRC patients is summarized in Table 2. CRC patients were divided into high-LTBP2 (*n* = 58) and low-LTBP2 (*n* = 146) groups according to the optimal cutoff value (180) of LTBP2 protein expression. High LTBP2 expression was obviously correlated with TNM stage (*χ*^2^ = 38.118, *P* < 0.001), T stage (*χ*^2^ = 15.953, *P* < 0.001), N stage (*χ*^2^ = 20.443, *P* < 0.001), and M stage (*χ*^2^ = 24.24, *P* < 0.001). Nevertheless, no significant correlations were observed between high LTBP2 protein expression and gender, age, tumor location, tumor differentiation, and preoperative CEA level.

### 3.4. Association between Survival, LTBP2 Protein Expression, and Clinical Features in CRC Patients

**Figure 3:**
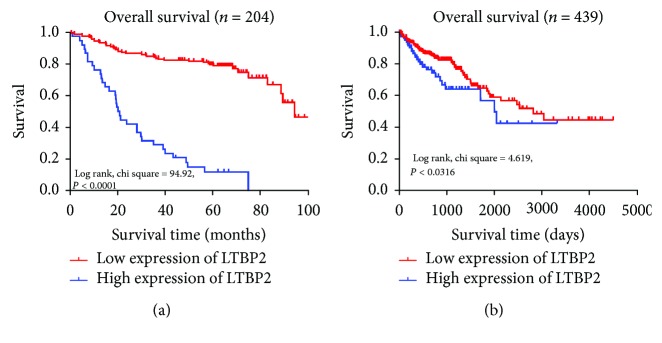
Survival curves of CRC patients by the Kaplan-Meier method and the log-rank test. (a) Patients with high LTBP2 expression (blue line) had significantly worse overall survival than those with low LTBP2 expression (red line). (b) In TCGA database, the high expression of LTBP2 (blue line) predicts poor overall survival for CRC patients.

204 CRC patients were followed up for a mean duration of 52.1 (range 0-100) months, and the 5-year overall survival (OS) rate was 55.9%. The Kaplan-Meier curve analysis showed that patients with high LTBP2 protein expression had an obviously shorter OS time than patients with low LTBP2 protein expression **(**log rank, *P* < 0.0001, Figure 3(a)), consistent with data from TCGA database (*P* = 0.0316, Figure 3(b)). Then, we analyzed the relationship between the OS rate and various prognostic factors in CRC patients using Cox regression univariate and multivariate analyses (Table 3). In the univariate analysis, LTBP2 protein expression (HR, 23.619, 95% CI: 13.036-42.794; *P* < 0.001), TNM stage (HR, 2.023, 95% CI: 1.532-2.672; *P* < 0.001), T stage (HR, 3.398, 95% CI: 1.692-6.825; *P* < 0.001), N stage (HR, 1.493, 95% CI: 1.207-1.847; *P* < 0.001), and M stage (HR, 5.983, 95% CI: 3.244-11.036; *P* < 0.001) were significantly correlated with OS. Afterwards, all the above factors were selected and put into the multivariate analysis to confirm whether LTBP2 protein expression was an independent factor of OS for CRC patients. Our results displayed that only LTBP2 protein expression (HR, 21.056, 95% CI: 11.274-39.326; *P* < 0.001) retained its significance and can be considered as an independent prognostic factor for OS of CRC patients (Table 3).

### 3.5. LTBP2 mRNA Expression in the CMS4 Subtype of CRC

**Figure 4:**
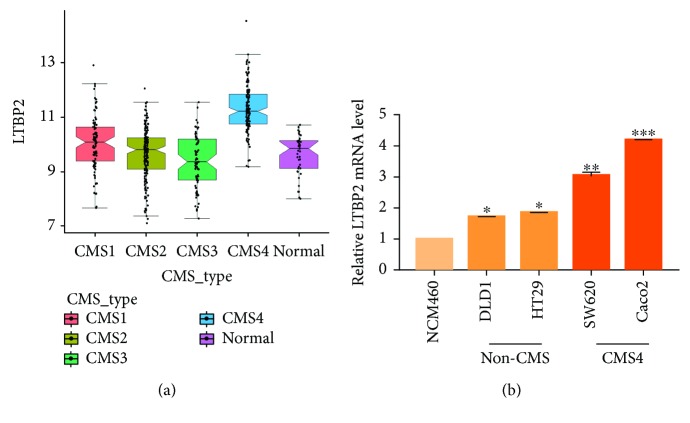
LTBP2 mRNA expression in the CMS4 subtype of CRC. (a) In TCGA database, LTBP2 mRNA expression was obviously higher in the CMS4 subtype than in other CRC subtypes (*P* < 0.0001). (b) LTBP2 mRNA expression was significantly higher in SW620 and Caco2 (CMS4) than in other CRC cell lines (non-CMS4) and in a normal colorectal epithelial cell (NCM460).

Further analysis of TCGA database showed that LTBP2 mRNA expression was especially higher in the CMS4 subtype than in other subtypes in 450 CRC samples (*P* < 0.0001, Figure 4(a)). It was confirmed in CRC cell lines, which means that LTBP2 mRNA expression was significantly higher in Caco2 and SW620 which better represent the CMS4 CRC cell line compared with other subtype CRC cell lines (DLD1 and HT29) and a normal colorectal epithelial cell line (NCM460) (Figure 4(b)).

## 4. Discussion

In the present study, we used qRT-PCR, TMA-IHC, and bioinformatic analyses to detect LTBP2 mRNA and protein expression levels in CRC and reveal the relationship between LTBP2 expression and the clinical features of CRC patients. Our results showed that LTBP2 mRNA expression was significantly higher in CRC tissues than in matched adjacent tissues. LTBP2 protein expression was also higher in CRC tissues than in matched tumor-adjacent tissues and benign colorectal diseases. High LTBP2 protein expression was obviously correlated with higher TNM stage, higher T stage, higher N stage, and higher M stage. High LTBP2 protein expression was related to poor overall survival in CRC patients and could be seen as an independent prognostic factor for CRC.

LTBPs are key factors in regulating TGF-*β* activities. On one hand, LTBPs covalently link to SL-TGF-*β* and then participate in folding, assembling, secretion, localization, and activation of TGF-*β* [14, 15]. On the other hand, LTBPs promote TGF-*β* storage by binding fibrillin microfibrils in the extracellular matrix protein [16]. However, LTBP2 is unique, which means that LTBP2 does not covalently combine with latent TGF-*β* and indirectly mediates TGF-*β* activities by competing with LTBP1 for binding fibrillin microfibrils [10, 17]. TGF-*β* plays a bidirectional role in the occurrence and development of tumors. When the tumor occurs, TGF-*β* is a tumor suppressor through its growth inhibition activity; but during the process of tumor development, TGF-*β* can promote cell invasion, metastasis, angiogenesis, and immunosuppression [18–20]. Therefore, it is not difficult to guess that LTBP2 also has bilateral effects towards tumor development. For example, LTBP2 was more downregulated in NPC tumor tissues than in matched normal tissues and played a suppressive role in tumor development and progression [21]. LTBP2 was also epigenetically silenced in chronic lymphocytic leukemia and melanoma [22, 23]. On the other side, LTBP2 was upregulated in head and neck squamous cell carcinoma and was significantly related to lymph node metastasis and pTNM stage [24]. Patients with high LTBP2 expression had poorer survival in pancreatic carcinoma [15]. A mechanism study displayed that knockdown of LTBP2 inhibited the proliferation, migration, invasion, and epithelial-to-mesenchymal transition (EMT) of a phenotype of thyroid carcinoma cells [25]. Furthermore, LTBP2 is both tumor suppressing and tumor promoting in ESCC, which means that LTBP2 was more downregulated in tumor tissues than in matched normal tissues, but high LTBP2 predicts poor overall survival [26]. Our results suggest that LTBP2 may act as an oncogene in CRC and may predict poor prognosis for CRC patients.

CMS4 is considered to be an aggressive CRC subtype with a characteristic of overexpressing genes involved in epithelial-to-mesenchymal transition (EMT), TGF-*β* signaling, angiogenesis, and extracellular matrix remodeling [4, 5]. The roles of TGF-*β* in CRC tumorigenesis are multivariate and controversial in literature. Some studies assume that the main role of TGF-*β* is in the tumor stroma owing to the absence of expression in malignant epithelium, whereas other reports point out that active TGF-*β* signaling can be examined in epithelial tumor cells [27, 28]. No matter what the exact mechanism of TGF-*β* is, the increased activity of TGF-*β* is related to prognosis and the presence of metastatic lesions [29], which can be partially explained by the ability of TGF-*β* to induce EMT [30]. EMT is a process correlated with poor disease outcome, and the activation of EMT is a remarkable feature of CMS4 [31]. Thus, TGF-*β* signaling, a known inducer of EMT [32], can be active in CMS4. Considering the LTBP2 expression in CRC and the strong association between LTBP2 and TGF-*β* signaling [33], we conjectured that LTBP2 could be a novel biomarker for CMS4. Thus, we further analyzed 450 CRC samples in TCGA database and divided them into 4 groups (CMS1, CMS2, CMS3, and CMS4). Interestingly, LTBP2 was found to be specifically higher in the CMS4 subtype and was also overexpressed in CMS4 CRC cell lines. Unfortunately, we did not classify the specimens according to CMS in our study and analyze the LTBP2 expression in the CMS4 subtype due to the current limitation of the clinical application.

Our study also has several other limitations. Firstly, our clinical samples were obtained only from Chinese patients; therefore, the results may not be representative of other CRC populations. Larger international studies will be necessary to validate our findings. Secondly, the detailed mechanism of LTBP2 in CRC (especially in the CMS4 subtype) has not been revealed. It is necessary for us to further investigate the role of LTBP2 in the CMS4 subtype of CRC in the future.

In conclusion, our study demonstrates that high LTBP2 expression correlates with inferior survival in patients with CRC and plays a critical role in the progression of colorectal cancer. High LTBP2 expression predicts a poor outcome for CRC patients and could be considered as a novel biomarker and potential therapeutic target for the high-risk CMS4 subtype of colorectal cancer.

## Figures and Tables

**Table 1:** LTBP2 protein expression in CRC tissues and other tissues.

Feature	*n*	LTBP2		*χ* ^2^	*P* value
		Low or no expression	High expression		
Chronic colitis	23	22 (95.7%)	1 (4.3%)		
Low-grade intraepithelial neoplasia	44	41 (93.2%)	3 (6.8%)		
High-grade intraepithelial neoplasia	22	17 (77.3%)	5 (22.7%)		
Cancer	204	146 (71.6%)	58 (28.4%)		
Surgical margin^a^	190	177 (93.2%)	13 (6.8%)		
Total	483	403 (83.4%)	80 (16.6%)	39.896	<0.001^∗^

^a^Epithelium without intraepithelial neoplasia from colorectal cancer. ^∗^*P* < 0.05.

**Table 2:** Correlation of LTBP2 protein expression with clinical characteristics of CRC patients.

Feature	*n*	LTBP2		*χ* ^2^	*P* value
		Low or no expression	High expression		
Total	204	146	58		
Gender				3.559	0.059
Male	127	85 (66.9%)	42 (33.1%)		
Female	77	61 (79.2%)	16 (20.8%)		
Age				0.001	0.976
≤60	63	45 (71.4%)	18 (28.6%)		
>60	141	101 (71.6%)	40 (28.4%)		
Location				1.343	0.511
Right colon	35	23 (65.7%)	12 (34.3%)		
Left colon	111	83 (74.8%)	28 (25.2%)		
Rectum	58	40 (69.0%)	18 (31.0%)		
Differentiation				0.095	0.954
Poor	4	3 (75.0%)	1 (25.0%)		
Well and middle	194	139 (71.6%)	55 (28.4%)		
Others^a^	6	4 (66.7%)	2 (33.3%)		
TNM stage				38.118	<0.001^∗^
0 and I	47	44 (93.6%)	3 (6.4%)		
II	78	60 (76.9%)	18 (23.1%)		
III	65	40 (61.5%)	25 (38.5%)		
IV	14	2 (14.3%)	12 (85.7%)		
T stage				15.953	<0.001^∗^
Tis, T1, and T2	54	50 (92.6%)	4 (7.4%)		
T3, T4	150	96 (64.0%)	54 (36.0%)		
N stage				20.443	<0.001^∗^
N0	128	104 (81.2%)	24 (18.8%)		
N1a	40	26 (65.0%)	14 (35.0%)		
N1b	20	10 (50.0%)	10 (50.0%)		
N2a,b	16	6 (37.5%)	10 (62.5%)		
M stage				24.24	<0.001^∗^
M0	190	144 (75.8%)	46 (24.2%)		
M1	14	2 (14.3%)	12 (85.7%)		
Preoperative CEA (ng/ml)				4.798	0.091
≤5	71	57 (80.3%)	14 (19.7%)		
>5	78	50 (64.1%)	28 (35.9%)		
Unknown	55	39 (70.9%)	16 (29.1%)		

^a^Mucinous adenocarcinoma, 6 cases. ^∗^*P* < 0.05.

**Table 3:** Univariate and multivariate analyses of prognostic factors for overall survival in CRC patients.

Variables	Univariate analysis			Multivariate analysis		
	HR	95% CI	*P* value	HR	95% CI	*P* value
Gender						
Male vs. female	1.591	0.966-2.619	0.068	NA		
Age						
≤60 vs. >60	1.008	0.0.617-1.648	0.973	NA		
Tumor location						
Right colon vs. left colon vs. rectum	1.038	0.773-1.393	0.806	NA		
Differentiation						
Poor vs. well and middle	0.533	0.177-1.607	0.264	NA		
TNM stage						
0 and I vs. II vs. III vs. IV	2.023	1.532-2.672	<0.001^∗^	0.980	0.469-2.050	0.958
T stage						
Tis, T1, and T2 vs. T3 and T4b	3.398	1.692-6.825	0.001^∗^	2.079	0.726-5.951	0.173
N stage						
N0 vs. N1a vs. N1b vs. N2a and N2b	1.493	1.207-1.847	<0.001^∗^	1.044	0.734-1.486	0.809
M stage						
M0 vs. M1	5.983	3.244-11.036	<0.001^∗^	1.986	0.665-5.934	0.219
Preoperative CEA (ng/ml)						
≤5 vs. >5	1.241	0.930-1.657	0.142	NA		
LTBP2 expression						
Low and none vs. high	23.619	13.036-42.794	<0.001^∗^	21.056	11.274-39.326	<0.001^∗^

Abbreviation: HR, hazard ratio; CI, confidence interval: NA, not considered in the multivariable model.

## Data Availability

The data used to support the findings of this study are available from the corresponding author upon request.
